# Exploring the role of pyroptosis in the pathogenicity of heart disease

**DOI:** 10.3389/fphys.2024.1357285

**Published:** 2024-04-05

**Authors:** Rohail Bhatti, Priscila Y. Sato

**Affiliations:** Department of Medicine, Division of Cardiovascular Disease, University of Alabama at Birmingham, Birmingham, AL, United States

**Keywords:** pyroptosis, heart disease, cell death, gasdermin, gasdermin D (GSDMD), gasdermin E (GSDME)

## Abstract

Cell death is an essential cellular mechanism that ensures quality control and whole-body homeostasis. Various modes of cell death have been studied and detailed. Unbalanced cell death can lead to uncontrolled cell proliferation (i.e., tumors) or excessive loss of cells (i.e., ischemia injury tissue loss). Thus, it is imperative for modes of cell death to be balanced and controlled. Here, we will focus on a recent mode of cell death called pyroptosis. While extensive studies have shown the role of this route of cell death in macrophages and monocytes, evidence for pyroptosis have expanded to encompass other pathologies, including cancer and cardiac diseases. Herein, we provide a brief review on pyroptosis and discuss current gaps in knowledge and scientific advances in cardiac pyroptosis in recent years. Lastly, we provide conclusions and prospective on the relevance to various cardiac diseases.

## Introduction

For a long time, cell death has been dismissed as an inevitable consequence of life. Yet, research has shown that this is a paramount process for organismal equilibrium as it is genetically encoded to eliminate damaged and harmful cells, maintaining harmonious balance within any tissue. According to recommendations of the nomenclature committee on cell death published in 2018 (Galluzzi et al., 2018), there are two main arms of cell death: a regulated cell death (RCD) and an accidental cell death (ACD). While the former poses an advantage for organismal homeostasis, the latter allows for an instantaneous and catastrophic demise of cells exposed to physical, chemical, or mechanical insults. RCD can occur in the absence of exogeneous environmental factors as a physiological built-in program or programmed cell death (PCD). RCD can also occur as an adaptive response to perturbations in the microenvironment that at least initially, aim at coping with stress and restore cellular equilibrium (Galluzzi et al., 2018). This type of RCD is important for homeostatic function as it eliminates useless or potentially harmful cells and allows for the release of damage-associated molecular patterns (DAMPs) in the extracellular milieu that may propagate the insult to neighboring cells.

In RCD, macroscopically cell death has been described to manifest itself in three different forms: type 1 or apoptosis, type 2 or autophagy, and type 3 or necrosis. Type 1 exhibits cell shrinkage, chromatin condensation, nuclear fragmentation, plasma membrane blebbing, and formation of small vesicles known as apoptotic bodies. These apoptotic bodies are efficiently taken up by neighboring cells with phagocytic activity and degraded within lysosomes. Type 2 is characterized by extensive cytosolic vacuole formation, culminating in phagocytic uptake and lysosomal degradation. Lastly, type 3 terminates by disposal of cell corpses in the absence of obvious phagocytic or lysosomal involvement (Schweichel and Merker, 1973; Galluzzi et al., 2007).

**FIGURE 1:**
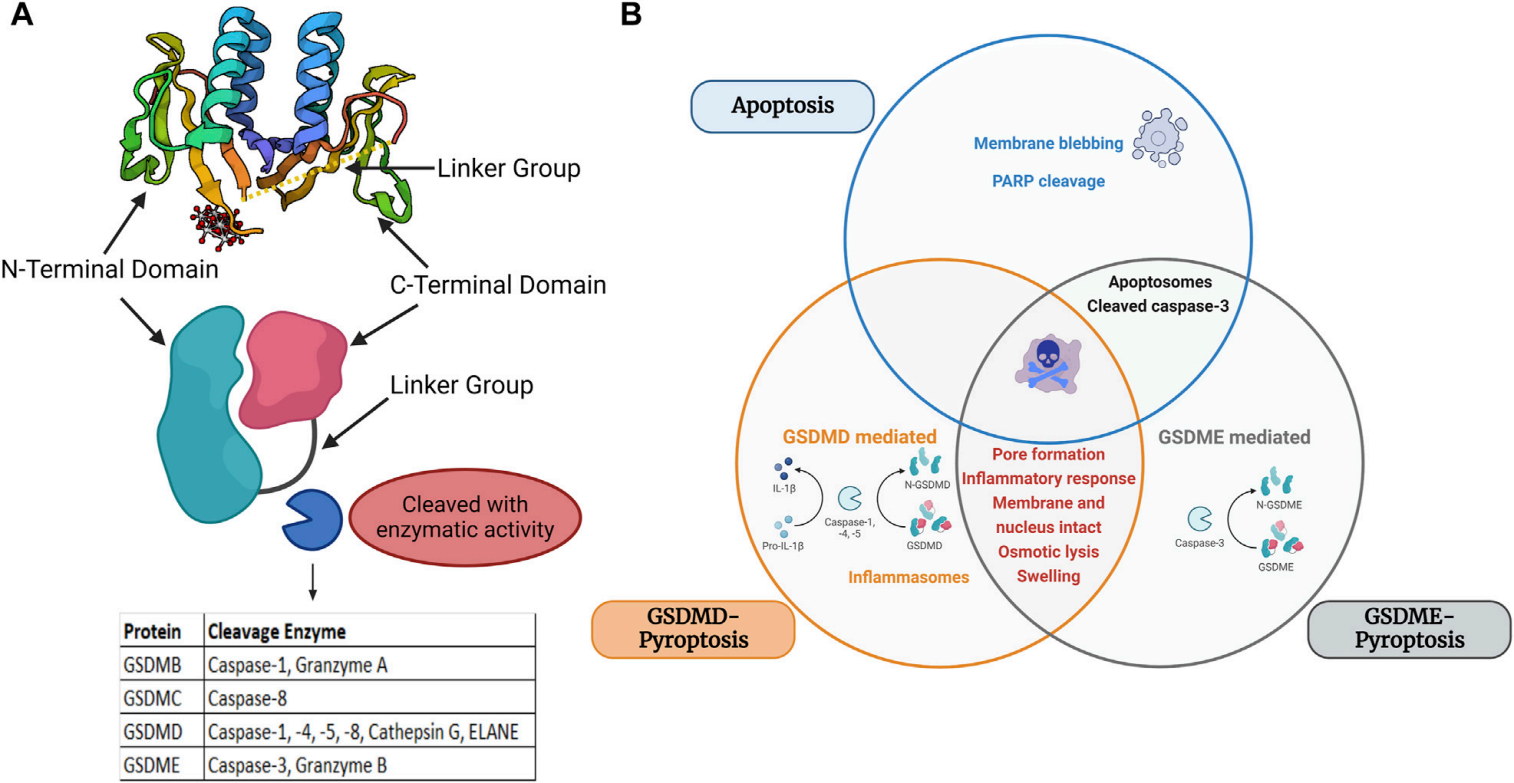
Structure and major domains of the gasdermin family members and Venn diagram of apoptosis, GSDME-pyroptosis, and GSDMD-pyroptosis. **(A)** Structural domains of gasdermins and cleavage area, Protein structure retrieved from Protein Data Bank (PDB) PDB ID: 6N90 **(B)** Overlapping and differential components of GSDMD/GSDME-pyroptosis and apoptosis are displayed in a Venn diagram. Apoptosis is marked by member blebbing and cleavage of poly (ADP-ribose) polymerase (PARP) and pyroptosis is marked by inflammatory response, osmotic lysis, and cell swelling. GSDME-mediated pyroptosis is mediated by caspase 3, whereas GSDMD is mediated by caspase 1/4/5/11. Image built with Biorender.

As the field continues to progress and new players and signaling pathways are identified in the orchestration of RCD, the interconnectivity between different modes of cell death has further complicated our understanding of beneficial *versus* pathological cell death responses. To date, there are at least 12 subroutes of major RCDs: intrinsic apoptosis, extrinsic apoptosis, MPT-driven necrosis (mitochondrial permeability transition driven necrosis), necroptosis, ferroptosis, parthanatos, entotic cell death, NETotic cell death (death by the formation of neutrophil extracellular traps (NETs)), lysosome-dependent cell death, autophagy-dependent cell death, immunogenic cell death, and pyroptosis (Galluzzi et al., 2018). We will focus this review on pyroptosis, a mode of cell death characterized by gasdermin-pore forming channels that leads to lytic cellular destruction (Figure 1). For detailed information on other sub routes of cell death please refer to Galluzzi et al., 2018.

## Pyroptosis discovery and cellular impact

The term pyroptosis is derived from the Greek word “pyro” for fire or fever and “ptosis” for falling (Cookson and Brennan, 2001). This form of RCD was first described as a form of pro-inflammatory PCD (Cookson and Brennan, 2001). Others have later also referenced as a form of necrotic cell death. As more research informs this mode of cell death, pyroptosis is mediated by gasdermin cell death (Galluzzi et al., 2018). The gasdermin superfamily in humans consists of six members: Gasdermin A, Gasdermin B, Gasdermin C, Gasdermin D, Gasdermin E, and DFNB59 (also known as PJVK (Pejvakin)) (Rogers and Alnemri, 2019). The gasdermin gene family was first reported in the early 2000s and are characterized as being pore-forming effector proteins that cause membrane permeabilization and lytic cell death. Except for PJVK/GSDMF, most GSDM family gene protein products contain three domains: the linker region, the GSDM C-terminal (GSDM-CT) domain, and the GSDM N-terminal (GSDM-NT) domain. The family has comparatively conserved N- and C-terminal domains, although each group has a unique linker region (Zou et al., 2021). All members share 45% homology and, except DFNB59, adopt a two-domain architecture composed of a globular N- and C- terminus with a flexible linker (Shi et al., 2015; Ding et al., 2016). It is within the flexible linker where cleavage occurs. The C-terminal domain entirely covers the NT hydrophobic pocket, it causes full-length GSDMs to be in an inhibited condition. The CT domain is made of α-helices to adopt a globular conformation. Proteolytic cleavage in the linker region releases both domains. The NT domain binds to negatively charged lipids in the plasma membrane once the C-terminal domain is dissociated. It enters the membrane, produces homo-oligomers, and undergoes significant conformational changes that allow for channel formation (Privitera et al., 2023) (Figure 1A).

Pyroptosis was first described in the 1980s as a caspase-1 dependent death in toxin-stimulated and pathogen-infected macrophages (Friedlander, 1986; Zychlinsky et al., 1992; Hilbi et al., 1997). Later the effect was expanded to position it as the main effector mechanism of pro-inflammatory caspases within the inflammasome complex. These caspases were found to cleave Gasdermin D within its central linker region (FLTF in humans and LLSD in mice) to generate a 31-KDa NT fragment and a 22-KDa CT fragment (He et al., 2015; Kayagaki et al., 2015; Shi et al., 2015; Chen et al., 2016). The N-terminus of GSDM proteins acts as an executor of pyroptotic cells, as it integrates with phosphatidylinositol phosphates, phosphatidylserine, and cardiolipin in cell membranes, oligomerizing to form a large 10–15 nm inner diameter pore (Ding et al., 2016; Liu et al., 2016). The N-terminus of all members except DFNB59, can oligomerize and form plasma membrane-spanning channels that drive disruption of ionic gradients, cell swelling, and cell death (Rogers et al., 2017; Rogers and Alnemri, 2019). This contrasts with apoptosis which is a noninflammatory and immunologically silent form of PCD (Rogers and Alnemri, 2019). Death receptor activation and apoptosome formation mediate apoptosis, which leads to membrane blebbing cell death of intact cells (Figure 1B).

**FIGURE 2:**
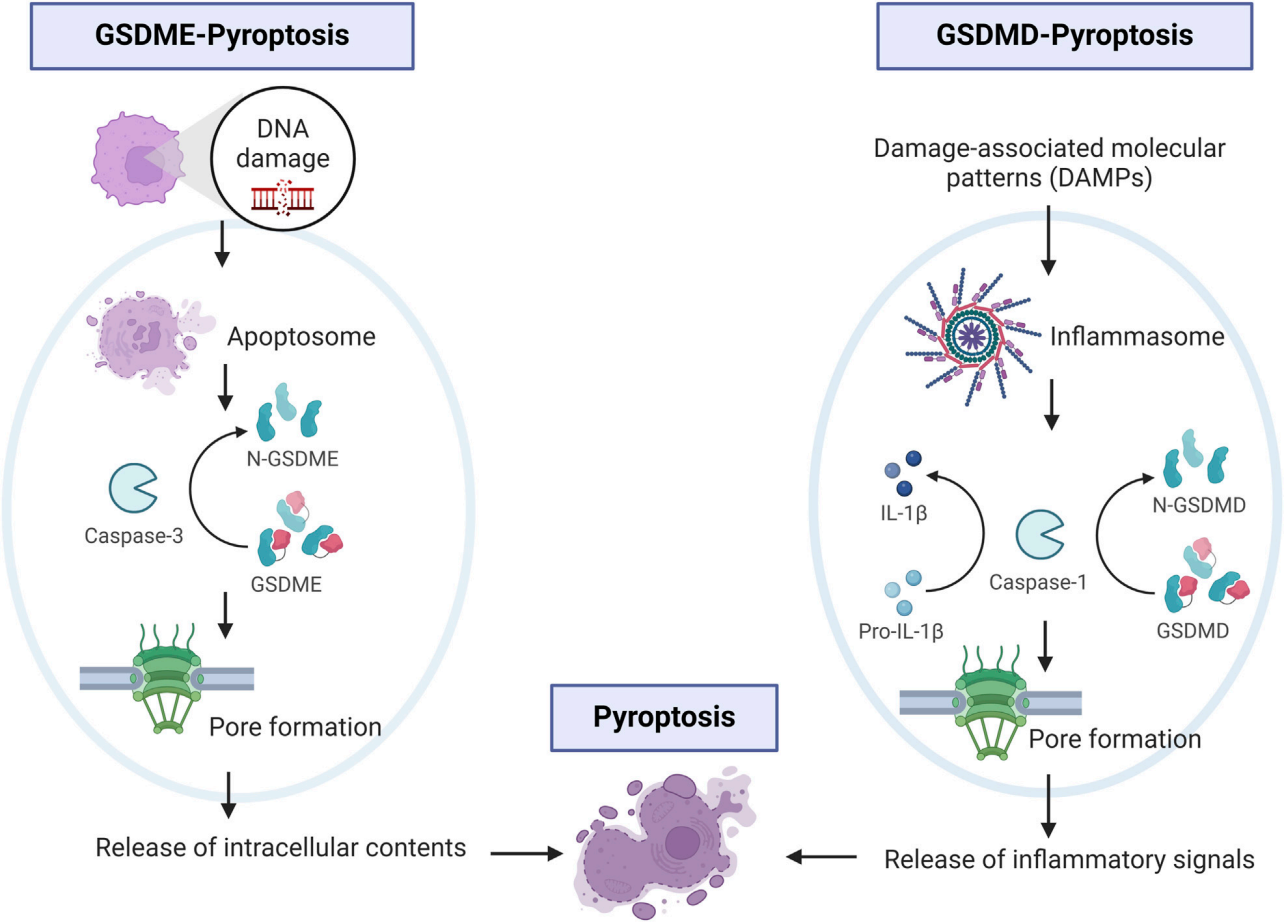
Schematic of main cellular signaling in GSDME- and GSDMD-pyroptosis. One left panel shows events of DNA damage that activate caspase-3 and subsequent cleavage of GSDME. On the right panel, cellular signaling involving DAMPs, activation of caspase-1 and cleavage of GSDMD leading to release of inflammatory signals. Image built with Biorender.

In 2015, three independent laboratories showed that GSDMD is a substrate for caspase −1,-4, −5, and (−11 in mice) (He et al., 2015; Kayagaki et al., 2015; Shi et al., 2015). Gasdermin-D mediated pyroptosis may occur via canonical and non-canonical signaling. In the canonical signaling, DAMPs or altered homeostasis activate inflammasome sensors, which activate caspase 1 leading to GSDMD cleavage and release of IL-1β and IL-18 (Bergsbaken et al., 2009; Zhou and Abbott, 2021). Assembly of the inflammasome is determined by activation of a unique pattern recognition receptor in response to pathogen-associated molecular patterns (PAMPs) or DAMPs (Shi et al., 2014; Liston and Masters, 2017). Inflammasomes are multi-protein assembles composed of three main parts: 1) sensor proteins (including NLRP1/3, NLRC4, AIM2, and pyrin recognizing PAMPs and DAMPs; 2) adaptor proteins containing a caspase recruitment domain (CARD) and apoptosis-associated speck-like proteins (ASC); and 3) pro-caspase 1 (Yu et al., 2021). Upon formation of the inflammasome, caspase-1 is activated and promotes the maturation of IL-beta and IL-18. In the non-canonical signaling, the non-canonical inflammasome also activates pyroptosis and GSDMD cleavage via caspase −4, −5 in humans (or the mouse ortholog −11) (Kayagaki et al., 2015; Silke and Vince, 2017). Caspase 4/5/11 are activated by directly recognizing cytosolic intracellular lipopolysaccharide (LPS) bound to the lipid A moiety (Shi et al., 2014). Activated caspase 4/5/11 directly cleave GSDMD, and lead to efflux of potassium (Ruhl and Broz, 2015; Aglietti et al., 2016). Potassium efflux stimulates the unfolding of Nod-like receptor pyrin domain containing 3 (NLRP3) inflammasomes which is postulated to promote maturation of interleukin (IL)-1β and IL-18 released from the pore (Figure 2) (Patil and Doshi, 2023). Munoz-Planillo et al., 2013 have shown in their study that NLRP3 inflammasomes are activated by extracellular Ca^2+^ via K^+^ efflux which is crucial for caspase-1 activity. GSDMD-mediated pyroptosis plays an important role in pathogen clearance and activation of the adaptive immune system.

Gasdermin-E mediated pyroptosis is mediated by activation of caspase 3 or the generation of cleaved caspase 3, which has been associated with secondary necrosis as it augments caspase-3 activation (Rogers et al., 2017; Rogers et al., 2019). It has been postulated that secondary necrosis results from insufficient and inefficient scavenging of apoptotic cells. As such, during the secondary necrosis phase, cells lose their membrane integrity, swell, and rupture releasing pro-inflammatory intracellular molecules, including IL-1β and HMGB1 (high mobility group box 1 protein) (Zhou and Abbott, 2021). Moreover, GSDME NT forms mitochondrial membrane-spanning pores which in addition, lead to the release of pro-apoptotic molecules (Rogers et al., 2017; Rogers et al., 2019) (Figure 2). Interestingly, GSDME is not the only mediator of secondary necrosis as deficiency of GSDME prevents secondary necrosis in some but not all cell types (Lee et al., 2018; Tixeira et al., 2018). This suggests additional GSDME-independent mechanisms of secondary necrosis. Studies investigating tissue expression showed GSDME in the cochlea, placenta, heart, brain, and kidney and GSDMD in immune cells, placenta, esophagus, and GI tract epithelium (Liu X. et al., 2021).

Overall, the abovementioned modes of cell death are responsive to specific cues and often described as separate entities, nevertheless often these modes happen in continuum exacerbating cellular death signaling that often, like many other cellular processes, intertwine and exert influence on one another.

## Pyroptosis: lessons learned from non-cardiac pathologies

Pyroptosis was first described as a caspase-1 dependent death in toxin-simulated or pathogen-infected macrophages (Friedlander, 1986; Zychlinsky et al., 1992; Hilbi et al., 1997).The term was coined by Cookson and Brennan to define a RCD that resembled apoptosis but was dependent on caspase 1 (Cookson and Brennan, 2001). Originally, pyroptosis was thought to be only relevant to the demise of monocytes or macrophages as it has a major role in innate immunity against intracellular pathogens (Zychlinsky et al., 1992; Bergsbaken et al., 2009). In fact, pyroptosis has recently taken center stage in various types of cancers and is considered the new frontier for cancer therapy. Alcohol accumulation has been linked to pyroptosis and the development of esophageal cancer (Wang F. et al., 2018). LPS-derived from gram-negative bacteria triggers pyroptosis via inflammasome activation and has been associated with carcinogenesis of Barrett’s esophagus (Nadatani et al., 2016). Pyroptosis is also associated with gastric cancer via downregulation of GSDMD accelerating expression of cdk2/cyclin A2 complexes that promote transition from S to G2 phase, thus accelerating gastric cancer cellular proliferation (Oakes et al., 2014; Wang W. J. et al., 2018). Diminished pyroptotic activity have been linked to tumor growth and proliferation of various other cancer types, including: ovarian cancer (Li et al., 2018), colorectal cancer (Ma et al., 2018; Tang et al., 2020), colon cancer (Derangere et al., 2014), cervical cancer (So et al., 2018), breast cancer (Pizato et al., 2018), and melanoma (Zhou et al., 2022) to name a few. Generally, pyroptosis activity suppresses tumor growth, thus gasdermin expression and activity have been postulated to be tumor suppressor genes. In fact, chemotherapeutic drugs in malignant mesothelioma have been shown to activate NLRP3 and caspase 1 (Westbom et al., 2015). Chemotherapeutics paclitaxel and cisplatin in lung cancer cells inhibit tumor growth by inducing GSDME-mediated pyroptosis (Zhang et al., 2019). Prognosis and drug response to melanoma have been associated with the expression pattern of pyroptosis-related genes (Zhou et al., 2022). Thus, in the oncology field the main therapeutic strategy is to induce cancer cell death. Hence, there is an active pursuit in strategies that re-activate gasdermins and induce pyroptotic signaling as a mechanism to slow down tumor growth and proliferation. Such is the strategy used in a recent study using raptinal to induce GSDME-dependent pyroptosis in therapy-resistant melanoma (Vernon et al., 2022). On-going clinical trials investigating the role of gasdermins in cancer are currently undergoing, including the analysis of GSDMD cleavage in cutaneous T-cell lymphoma (NCT05333367), and another is investigating GSDMD and inflammasome activation in acute myeloid leukemia patients (NCT06200441).

Unlike in cancer, other pathologies have shown that pyroptotic activity is involved in the development of disease progression. Pyroptosis has been linked to lipid peroxidation in lethal polymicrobial sepsis (Kang et al., 2018), covid infection (Ferreira et al., 2021), progression of multisystem inflammatory disease (Xiao et al., 2018), and required for severe disease during enterovirus infection (Dong et al., 2022). This includes the immunogenic role of GSDMD-pyroptosis in neutrophils or NETosis, where cleavage of GSDMD results in release of NETs. Thus, fine-tuning of molecular mechanisms driving pyroptosis are paramount for physiological process and key to promote healthy cellular environments. Current on-going clinical trials involving pyroptotic signaling and sepsis include NCT06013033 and NCT05914428. Thus far, GSDME mutations are the only reported human gasdermin mutations linked to human pathologies, as is the case in autosomal dominant non-syndromic hearing loss (Van Laer et al., 1998). Although GSDME is expressed in multiple tissues, it remains unclear why in these patients carrying the mutation, the cochlea is the major tissue impacted.

## Pyroptosis in the heart

The impact of pyroptosis in the heart has lagged other fields but has been the topic of recent investigations. Cell death plays an important role in cardiac biology, particularly during stress and ischemic insults (Chen et al., 2013; Fan et al., 2013; Sato et al., 2018). Studies have shown that renewal of adult cardiomyocytes is low, hence excessive cellular death detrimentally impact myocardial functional outcomes. Recent work has shown a pivotal role for GSDMD in cardiomyocytes post-myocardial ischemia-reperfusion (IR) injury. Using a cardiac specific GSDMD-knockout, Shi et al., 2015 showed that GSDMD is involved in IR injury and that inhibition of this mechanism decreases IR injury. Interestingly, in these IR studies GSDME levels did not change suggesting that in IR inflammatory pyroptosis in adult cardiomyocytes may be more prominent than apoptotic signaling. Potentially this could be because studies were relatively short-term post-reperfusion. In a murine model of acute myocardial infarction (AMI), transcriptome analysis showed that the immune system was activated at 6 h post-MI while pyroptosis was activated 24 h post-AMI. Using VX-765, an inhibitor of pyroptosis, the same study showed a reduction in infarct size and improved cardiac function (Liu W. et al., 2021). Studies investigating the cardioprotective role of GDF11 (Growth Differentiation Factor 11) post-AMI revealed the upregulation of transcription factor HOXA3, a regulator of NLRP3 formation, thus linking GDF11 as a negative regulator of cardiac pyroptosis (Li et al., 2020). GSDMD-pyroptotic signaling has also been shown to play a role in cardiac autophagy and doxyrubicin-induced cardiotoxicity (Qu et al., 2022; Ye et al., 2022). Moreover, GSDMD has been implicated in pathological cardiac hypertrophic mechanisms in response to Angiotensin II stimulation (Han et al., 2022). GSDME-mediated pyroptosis has been linked to the progression of atherosclerosis, where macrophage pyroptosis and inflammation are suppressed when GSDME is blocked (Wei et al., 2023). In another murine model, a type I transmembrane protein called CXADR-like membrane protein (CLMP), a member of the CTX family, prevents the heart from experiencing severe pyroptosis after an MI. However, it also mentions that the protein’s expression is controlled because elevated expression can have detrimental consequences (Han et al., 2020). Thus, multiple studies currently support the notion that pyroptosis activation induces signaling that is detrimental to myocardial function.

Excitingly to the field, recent studies in patients with acute myocardial infarction (AMI) have shown an elevated expression of GSDMD in peripheral blood mononuclear cells (PBMCs) (Weng et al., 2022). Likewise, GSDMD is required for enhanced mobilization of neutrophils to the infarcted heart. The activated NLRP3 inflammasome generated after AMI leads to further myocardial damage, both directly and indirectly, by promoting inflammatory cell death by production of IL-1β through pyroptosis (Toldo and Abbate, 2018). Loss of GSDMD resulted in attenuated IL-1β release from neutrophils with decreased neutrophils and monocytes to the infarcted heart (Jiang et al., 2021). Important to note, these latter studies were performed in a global model of GSDMD knockout model, where it can be argued that given the cardiac-specific GSDMD KO results (Shi et al., 2021), there could be decreased pyroptosis in adult cardiomyocytes which could contribute to the reduced recruitment of PBMC-derived cells. Jiang et al. showed that genetic knockout and pharmacological inhibition of GSDMD improved cardiac function after AMI (Jiang et al., 2022). Zhong et al. identified GSDMD inhibitor Y1 (GI-Y1) as a selective GSDMD inhibitor with cardioprotective properties using pharmacological screening, structure-based virtual screening, and subsequent pharmacological validations (Zhong et al., 2023).

Atherosclerosis, a chronic inflammatory disease impacting the walls of the arteries, is a main contributor of cardiovascular disease and mortality. Huang et al., 2024 recent work show that the genetic elimination of GSDMD delays the progression of atherosclerosis. This finding confirms that GSDMD plays a crucial role in the pyroptosis of endothelial cells and macrophages by forming neutrophil extracellular traps (NET) and releasing inflammatory factors. Another study suggested that Wilms tumor 1-associated protein, an element of the m6A methyltransferases linked to cardiac sympathetic hyperactivity, increases pyroptosis and inflammation in endothelial cells involved in exacerbating atherosclerosis by the NF-κB/NLRP3 mediated inflammatory signaling pathway (Hu et al., 2024; Huang et al., 2024). Khojali et al., 2024 has also highlighted non-coding RNAs as an important component in the progression of atherosclerosis at the molecular level. These results suggest that GSDMD could be a potential therapeutic target for the management of atherosclerosis.

Heart failure (HF) is an outcome of cardiac damage due to several cardiomyopathies like MI, hypertension, and ischemic heart disease. HF is associated with cardiac remodeling which causes abnormalities in the structure and functionality of the heart. Pyroptosis is known to impact fibrotic, hypertrophic, and inflammatory stages. Drugs that inhibit COX (cyclooxygenase) and the formation of reactive oxygen species impact the NRLP3 pathway and suppressing Akt/GSK3β/NF-κB/NLRP3 signaling have shown promising results in the treatment post-MI (Habimana et al., 2022). Several pre-clinical therapeutic strategies targeting the NLRP3 inflammasomes, Caspase-1, IL-1β, and GSDMD N-terminal domains have investigated showing improvement in cardiovascular performance (Chai et al., 2022). Recent bioinformatics analysis of pyroptosis related genes (PRGs) in cardiovascular diseases have highlighted potential pharmacological targets within this pathway. A bioinformatics analysis by Tang et al., 2024 has investigated the molecular mechanisms underlying the functions of the PRGs in hypertrophic cardiomyopathy, an inherited cardiovascular disease characterized by left ventricular hypertrophy, myocardial hypercontraction, cardiomyocyte disruption, and fibrosis. They identified and validated key biomarkers in this regard and opened new avenues for potential pharmacological targets within pyropoptic signaling. Yu et al., 2024 identified PRGs in calcific aortic valve disease where as putative pharmacological targets for the treatment of calcified valve diseases.These potential targets in cardiovascular diseases highlight the significance of pyroptosis in the development of HF and identify novel opportunities for pharmacological interventions and targeting. Overall, cardiac studies converge on the important role of pyroptosis signaling in the heart. Yet, apoptotic signaling, GSDME, and crosstalk between GSDME and GSDMD in the heart remain less understood. To our knowledge, there are no currently on-going clinical trials targeting inhibition of pyroptosis in myocardial ischemic injury or other cardiac abnormalities.

## Conclusions and therapeutic potential for the treatment of cardiac pathologies

Recent studies have contributed to substantiate the impactful breadth that pyroptosis has to overall physiology involving various cell types. While pyroptosis is currently pursued as a mode for tumor regression and decrease in proliferation, cautious pharmacological strategies need to extrapolate the possibility for a negative impact to the heart and other biological systems. In the other hand, strategies that envision inhibition of pyroptosis as a mode for cardiac protection need to be zealous for a possible increased susceptibility of tumor formation. Although pyroptosis is a significant and important mechanism for cell viability and health, studies suggest a small window for “normal” gasdermin signaling. Given current strategies in the oncology field exploring activation of pyroptosis, understanding of how pyroptosis plays a role in the heart is essential to our understanding of cardiotoxicity and will inform novel strategies to increase lifespan in cancer patients. Both arms of investigation, in the cancer and cardiac field, are paramount for greater understanding of the associated pathophysiology to better develop treatments for these patients.
